# Human Placenta-Derived Adherent Cell Treatment of Experimental Stroke Promotes Functional Recovery after Stroke in Young Adult and Older Rats

**DOI:** 10.1371/journal.pone.0086621

**Published:** 2014-01-21

**Authors:** Amjad Shehadah, Jieli Chen, Ajai Pal, Shuyang He, Andrew Zeitlin, Xu Cui, Alex Zacharek, Yisheng Cui, Cynthia Roberts, Mei Lu, Robert Hariri, Michael Chopp

**Affiliations:** 1 Department of Neurology, Henry Ford Hospital, Detroit, Michigan, United States of America; 2 Celgene Cellular Therapeutics, Warren, New Jersey, United States of America; 3 Department of Biostatistics and Research Epidemiology, Henry Ford Hospital, Detroit, Michigan, United States of America; 4 Department of Physics, Oakland University, Rochester, Michigan, United States of America; University of Münster, Germany

## Abstract

**Background:**

Human Placenta-Derived Adherent Cells (PDAC®) are a novel mesenchymal-like cell population derived from normal human placental tissue. PDA-001 is a clinical formulation of PDAC® developed for intravenous administration. In this study, we investigated the efficacy of PDA-001 treatment in a rat model of transient middle cerebral artery occlusion (MCAo) in young adult (2–3 month old) and older rats (10–12 months old).

**Methods:**

To evaluate efficacy and determine the optimal number of transplanted cells, young adult Wistar rats were subjected to MCAo and treated 1 day post MCAo with 1×10^6^, 4×10^6^ or 8×10^6^ PDA-001 cells (i.v.), vehicle or cell control. 4×10^6^ or 8×10^6^ PDA-001 cells were also tested in older rats after MCAo. Treatment response was evaluated using a battery of functional outcome tests, consisting of adhesive-removal test, modified Neurological Severity Score (mNSS) and foot-fault test. Young adult rats were sacrificed 56 days after MCAo, older rats were sacrificed 29 days after MCAo, and lesion volumes were measured using H&E. Immunohistochemical stainings for bromodeoxyuridine (BrdU) and von Willebrand Factor (vWF), and synaptophysin were performed.

**Results:**

In young adult rats, treatment with 4×10^6^ PDA-001 cells significantly improved functional outcome after stroke (p<0.05). In older rats, significant functional improvement was observed with PDA-001 cell therapy in both of the 4×10^6^ and 8×10^6^ treatment groups. Functional benefits in young adult and older rats were associated with significant increases in the number of BrdU immunoreactive endothelial cells, vascular density and perimeter in the ischemic brain, as well as significantly increased synaptophysin expression in the ischemic border zone (p<0.05).

**Conclusion:**

PDA-001 treatment significantly improved functional outcome after stroke in both young adult and older rats. The neurorestorative effects induced by PDA-001 treatment may be related to increased vascular density and synaptic plasticity.

## Introduction

Cell-based therapies currently being evaluated for stroke treatment include neural stem and progenitor cells, cord blood, and bone marrow-derived mesenchymal stromal cells (MSCs) [1]–[4]. Bone marrow-derived MSCs (BMSCs) have been extensively studied in animal models of stroke [5]–[7] and have shown promising therapeutic potential in myocardial [8], limb [9], and brain ischemia [10]. However, BMSCs must be obtained through an invasive procedure, are rare in the adult human bone marrow [11], and their number significantly decreases with the age of the individual [12]. On the other hand, the placenta is a rich source of stem cells, no invasive procedures are needed to obtain the organ, and there are no ethical concerns regarding their use. Additionally, placenta-derived adherent stromal cells have multi-lineage differentiation potential similar to BMSCs in terms of morphology, cell-surface antigen expression, and gene expression patterns, are able to differentiate into many types of cells, are easy to isolate, and large amounts of MSCs can be obtained in culture [8]–[10].

Placenta-Derived Adherent Cells (PDAC®) are mesenchymal stromal-like cells isolated from human placental tissue and culture-expanded [13]–[15]. PDAC® display the nominal phenotype CD34−, CD10+, CD200+, and CD105+ [16]. These cells are exclusively of placental, non-maternal origin and are karyotypically normal. Like MSCs derived from bone marrow and other sources, PDAC® demonstrate immunomodulatory properties in vitro and in animal models. PDAC® suppress T-cell proliferation and exhibit immunomodulatory effects on other cell types such as macrophages, dendritic cells and T-cell subsets [13].

PDA-001 (cenplacel-L) is a formulation of PDAC®, developed for intravenous administration and is currently being assessed as a treatment for Crohn's disease [15].

Previously, Kranz et al. [17] showed that double infusion of placenta derived MSCs (1×10^6^ cells) at 8 hours and 24 hours improved functional outcome in experimental stroke, however, a single injection at 24 hours did not improve outcome. Additionally, a recently published study by Yu et al. [18] showed that transplantation of 1×10^6^ stem cell-like placenta cells improved functional recovery when administered at 3 hours after stroke in dogs. In our previous study, we demonstrated that PDA-001 treatment improves functional outcome in young adult rats when administered 4 hours after middle cerebral artery occlusion (MCAo) [19]. However, treatment administration within four hours after stroke may not always be feasible in clinical practice. Furthermore, to maximize successful translation of an effective therapy from the lab to the clinic, efficacy of treatment should also be demonstrated in older animals [20]–[22]. Therefore, in the present study, we investigated the efficacy of PDA-001 treatment when administered 24 hours after MCAo in both young adult and older rats.

## Materials and Methods

This study was carried out in strict accordance with the recommendations in the Guide for the Care and Use of Laboratory Animals of the National Institutes of Health. The protocol was approved by the Henry Ford Health System's Institutional Animal Care and Use Committee (IACUC approval number: 0889). All surgery was performed under isoflurane anesthesia, and all efforts were made to minimize suffering.

### PDA-001 preparation

PDA-001, provided by Celgene Cellular Therapeutics [6], [13], was removed from the shipping container and placed in a ziploc bag before being thawed in a 37°C water bath. Once thawed, the cell suspension was removed from the cryobag with a 10 ml syringe fitted with an 18G needle. The cell suspension was mixed using a 10 ml pipette. To determine cell concentration, 900 µl of Dextran solution and 100 µl of cell suspension were mixed, after which 200 µl of this dilution was mixed with 200 µl of trypan blue. Ten microliters of this final dilution (20×) was counted on a hemocytometer before each injection to calculate cell viability. The average viability was 96.9%±2.0. The cells were then diluted to the desired concentrations of 1, 4, or 8×10^6^ cells/1–2 ml.

### Animal middle cerebral artery occlusion (MCAo) model

#### Young adult rats

Young adult male Wistar rats (270–300 g, 2–3 months) were subjected to 2 hours of transient right MCAo induced by advancing a 4-0 surgical nylon suture (18.5–19.5 mm, as determined by the animal weight) with its tip rounded by heating near a flame, to block the origin of the MCA, using a method of intraluminal vascular occlusion modified in our laboratory [23], [24]. Rectal temperature was maintained at 37°C throughout the surgical procedure using a feedback regulated water heating system.

To determine the optimal number of transplanted PDA-001 cells for treatment of stroke in young adult rats, at 1 day post MCAo, randomly selected animals were injected, via a tail vein, with either: vehicle control (Dextran, n = 9), cell control (4×10^6^ human dermal fibroblast cells (FBC) in 2 ml Dextran, n = 10), or PDA-001 cells (1×10^6^, n = 10; 4×10^6^, n = 10; or 8×10^6^, n = 13) in Dextran. All rats were sacrificed at 56 days after MCAo.

#### Older rats

Older male Wistar rats (600–750 g, 10–12 months) were subjected to 90 minutes of MCAo induced by advancing a surgical 3-0 nylon with its tip rounded by heating near a flame, to block the origin of the MCA, using the method of intraluminal vascular occlusion described above.

To test the efficacy of PDA-001 treatment in older rats, at 1 day after MCAo, randomly selected animals (n = 12 per group) were injected, via a tail vein, with vehicle control (cell media), or with PDA-001 (4×10^6^ or 8×10^6^ cells). All rats were sacrificed at 29 days after MCAo.

### Bromodeoxyuridine labeling

In order to identify newly synthesized DNA for identification of cell proliferation in ischemic brain, rats received injections of bromodeoxyuridine (BrdU, Sigma Chemical, 50 mg/kg in 0.007 N NaOH physiological saline, Sigma, St. Louis MO), intraperitoneally (i.p.) daily for 14 days after MCAo.

### Functional tests

For each young adult rat, functional tests were performed 24 hours after MCAo (immediately before PDA-001 therapy) and at 7, 14, 21, 28, 42, and 56 days.

For each older rat, functional tests were performed 24 hours after MCAo (immediately before the PDA-001 therapy) and at 3, 7, 14, 21 and 28 days after MCAo.

All functional tests were performed by an investigator adequately trained in functional measurements and blinded to the experimental groups.

The battery of functional tests (for young and older animals) consists of an Adhesive-removal somatosensory test, a modified Neurological Severity Score (mNSS) and a Foot-fault test.

#### Adhesive-removal somatosensory test

The brain undergoes dynamic change after stroke, and animal behavior is altered depending on the stage of recovery. The adhesive-removal test uses an adhesive dot as a bilateral tactile stimulus on the distal-radial region of the wrist of each forelimb. The time for each rat to remove each adhesive stimulus from its forelimbs was recorded from 5 trials per day. Individual trials were separated by at least 5 min. The adhesive-removal test is sensitive in an early stage of somatosensory recovery, but stabilizes after one month [25], [26]. However, using a smaller adhesive tab increases the sensitivity for long term functional outcome after stroke. Therefore, in the initial testing and up to one month after MCAo, two small adhesive-backed paper dots (of equal size, 113.1 mm^2^) were used; smaller dots (56.6 mm^2^) were used thereafter to increase the sensitivity. All rats were familiarized with the testing environment before surgery and were returned to their cages after each testing session.

#### Modified neurological severity score (mNSS)

The mNSS is a composite of motor, sensory, balance and reflex tests and is graded on a scale of 0 to 18 (normal score 0; maximal deficit score 18). One score point is awarded for the inability to perform the test or for the lack of a tested reflex; thus, a higher score indicates a more severe injury [3], [5].

#### Foot-fault test

The Foot-fault test assesses placement dysfunction of forelimbs [27], [28]. Animals were placed on an elevated grid floor (45 cm×30 cm), 2.5 cm higher than a solid base floor, with 2.5 cm×2.5 cm diameter openings. Animals tend to move on the grid with their paws placed on the wire frame. When animals inaccurately place a paw, the front limb falls through one of the openings in the grid. When the paw falls through or slips between the wires, it is recorded as a foot fault. A total of 100 of steps (movements of each forelimb) were counted, and the total number of foot faults for the left forelimb was recorded. The percentage of foot faults of the left paw out of total steps was calculated.

### Histological and immunohistochemical assessment

Rats were anesthetized with Ketamine (80 mg/kg) and Xylazine (13 mg/kg) via i.p. injection and the depth of anesthesia was monitored by paw pinch reflex. The animals were then subjected to cardiac puncture with saline (approximately 200 ml/rat) perfusion and then 4% paraformaldehyde (approximately 50 mL/rat) before brains were fixed and embedded in paraffin.

Each brain was cut into 2 mm thick coronal blocks, for a total of 7 blocks per animal. The brain tissue was processed, embedded, and 6 µm thick paraffin coronal sections from each block were cut and stained with hematoxylin and eosin (H&E) for calculation of lesion volume [29]. The indirect lesion area, the intact area of the ipsilateral hemisphere subtracted from the area of the contralateral hemisphere, was calculated using the Global Lab Image analysis system (Data Translation, Marlboro, MA) [29]. Lesion volume is presented as the percentage of the ipsilateral indirect lesion volume compared to the contralateral hemisphere.

### Immunohistochemical staining

The brain sections obtained from rats treated with the optimal number of PDA-001 cells in young adult rats, vehicle and cell control groups were used for immunostaining. For older rats, brain sections obtained from PDA-001 treatment groups and vehicle control group were used for immunostaining.

A standard paraffin block was obtained from the center of the lesion (bregma −1 mm to +1 mm). A series of 6 µm thick sections were cut from the block. Three coronal brain sections were used for each immunohistochemical staining with antibodies against BrdU (a proliferating cell marker, 1∶100, Boehringer Mannheim, Indianapolis, IN), von Willebrand Factor (vWF, 1∶400; Dako, Carpenteria, CA), and synaptophysin (Boehringer Mannheim Biochemica, Monoclonal antibody, clone SY 38, 1∶40). The immunostaining analysis was performed by an investigator blinded to the experimental groups.

#### Quantitation

For measurement of vascular density and perimeters, the vWF immunostained coronal section was digitized under a 20× objective via the MCID computer imaging analysis system (Imaging Research, St. Catharines, Canada). The total perimeter of ten enlarged thin walled vessels, and number of vessels located in the ischemic boundary zone (IBZ) were measured. The total number of vessels was divided by the total tissue-area to determine vascular density. Data are presented as total number of vessels of per mm^2^.

For measurement of endothelial cells proliferation, BrdU immunostained sections were digitized under a 40× objective via the MCID computer imaging analysis system. BrdU positive endothelial cells within a total of 10 enlarged and thin walled vessels located in the IBZ were counted in each section.

To measure synaptophysin immunoreactivity, the immunostained coronal section and eight fields of view from the ischemic penumbra (cortex and striatum) in each section were digitized under a 20× objective. The positive immunoreactive area was measured using the MCID computer imaging analysis system. The data are presented as a percentage of positive immunoreactivity area in the ischemic border.

### Statistical Analysis

The effects of PDA-001 on the functional recovery in young and older adult rats were measured from the three behavior tests. The data were evaluated for normality. Data transformation was performed if data were not normal. Behavioral outcomes were not normal; hence, ranked data were used for the analysis.

The global test using the Generalized Estimating Equation (GEE) [30] analyzed the group difference in functional recovery measured from multiple behavior tests. The pair-wise treatment comparison was conducted if the overall treatment effect was detected at the 0.05 level. If a global test was significant at the 0.05 level, the group difference was conducted on each functional test at the 0.05 level. Otherwise, the pair-wise treatment comparison or the treatment comparison on each functional outcome was considered exploratory. The global test on multiple outcomes is more efficient than on a single outcome when the group effects are consistent for all the outcomes (e.g., the positive correlation). The correlation among three outcomes is estimated using the GEE.

In young adult rats, we first tested the difference between the two control treated groups (FBC-cell control group and Dextran vehicle control), and the control data were combined if no difference was detected. A separate analysis was also conducted using Dextran vehicle control treated rats. Data are presented as mean ± SE (standard error).

One-way ANOVAs were used to test for differences in histology measures among the treatment groups. The pair-wise group comparison was conducted at the 0.05 level if the overall group effect was observed at 0.05 level. Otherwise, the pair-wise group comparison was considered exploratory.

Spearman or Pearson correlation coefficients were calculated among the histological evaluation measurements and their correlation with the functional recovery stratified by the treatment groups.

## Results

### Neurological outcome and lesion volume

To determine the optimal number of transplanted cells, young adult Wistar rats were subjected to MCAo and treated 24 hours after MCAo with 1×10^6^, 4×10^6^ or 8×10^6^ PDA-001 cells (i.v.) and functional outcome was compared to vehicle and cell controls. Functional response using three testing methods (Adhesive-removal somatosensory test, modified Neurological Severity Score (mNSS) and Foot- fault test) was measured before PDA-001 treatment and at 7, 14, 21, 28, 42, and 56 days after MCAo. The global test using the Generalized Estimating Equation (GEE) was performed to detect the differences in functional recovery among the groups. Because there was no significant difference between the two control groups (Dextran vehicle control and FBC-cell control, p-values >0.22), the two controls were combined for comparison to PDA-001 treatment.

**Figure 1:**
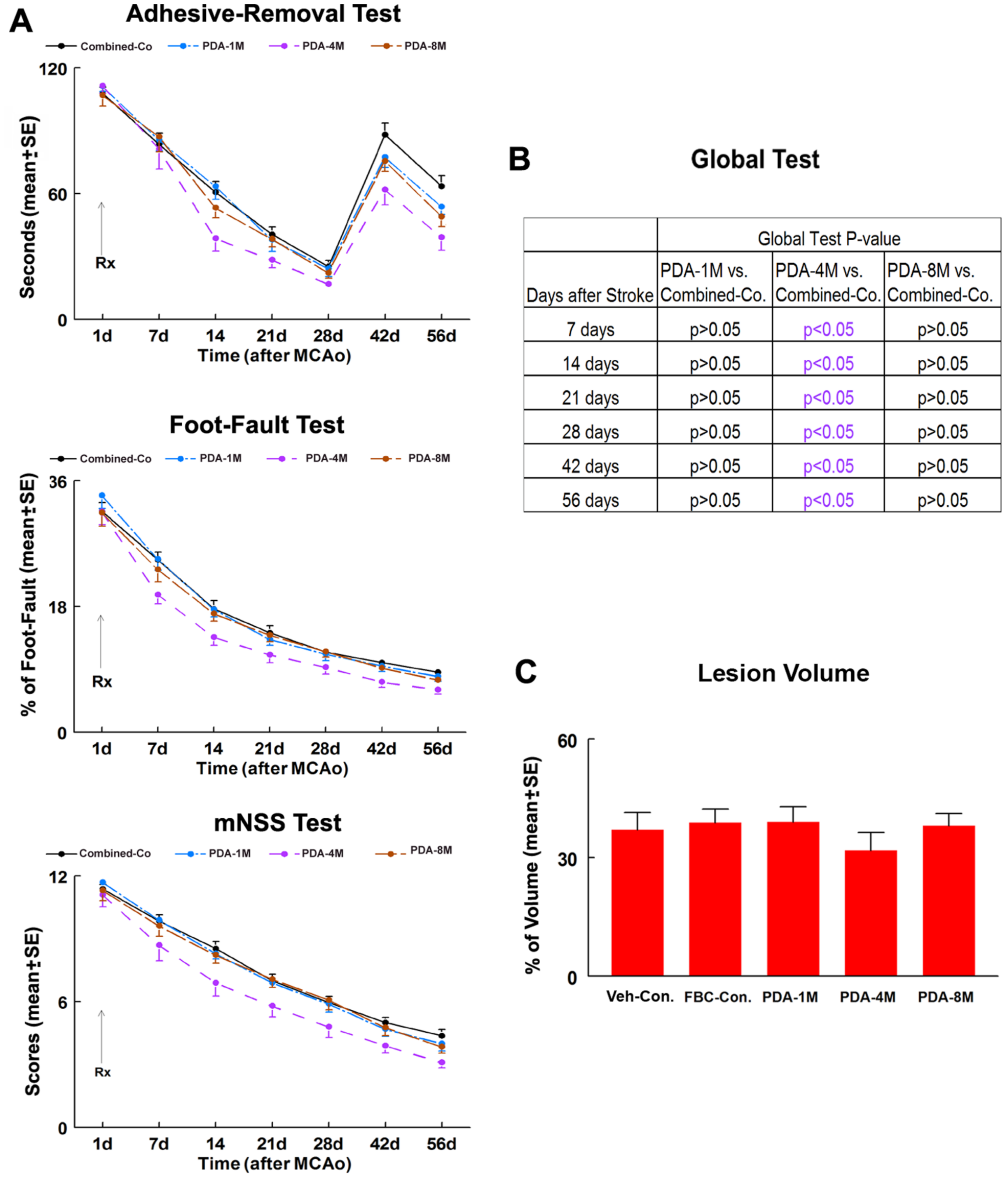
Neurological outcome and lesion volume in young adult rats. Panel **A** shows adhesive-removal, foot-fault and mNSS tests after stroke in the 4 young adult rats experimental groups (combined-control, 1×10^6^, 4×10^6^, and 8×10^6^ PDA-001 treatment groups). Panel **B** shows the global test analysis to detect the differences in functional recovery among the 4 groups. Panel **C** shows the lesion volume in the 5 young adult rats experimental groups. Error bars represent standard error of the mean (mean±SE).

**Figure 2:**
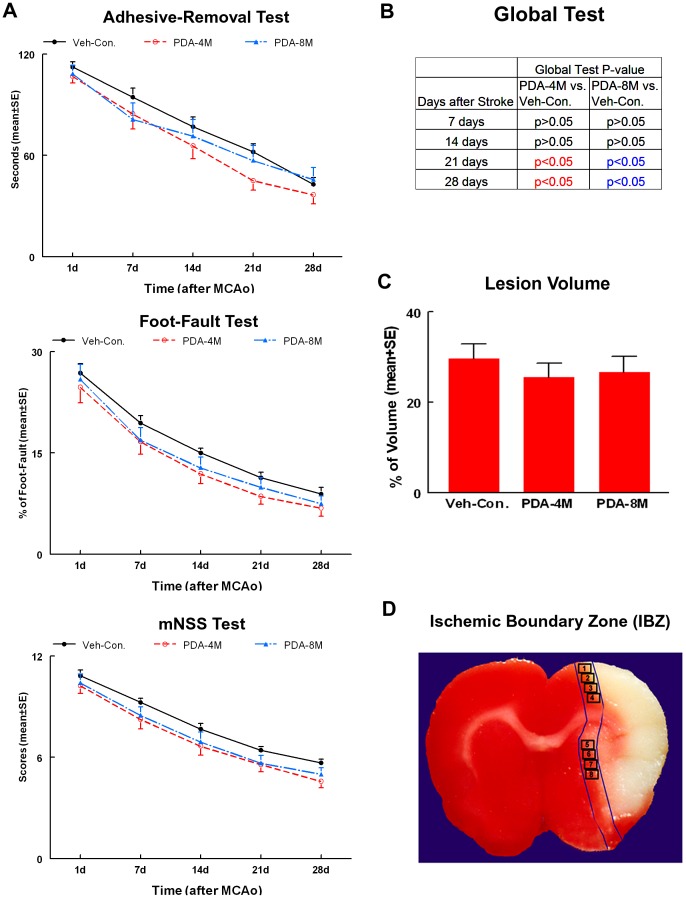
Neurological outcome and lesion volume in older rats. Panel **A** shows adhesive-removal, foot-fault and mNSS tests after stroke in the 3 older rats experimental groups (vehicle-control, 4×10^6^ and 8×10^6^ PDA-001 treatment groups). Panel **B** shows the global test analysis to detect the differences in functional recovery among the 3 groups. Panel **C** shows the lesion volume in the 3 older rats experimental groups. Panel **D** shows the ischemic boundary zone (IBZ). Error bars represent standard error of the mean (mean±SE).

Young adult rats treated with the lowest (1×10^6^) and highest (8×10^6^) number of PDA-001 cells did not show significant improvement in functional outcome compared to the combined-control group (**Figure 1A–B**). However, rats treated with 4×10^6^ PDA-001 cells showed significant improvement in functional recovery compared to combined-control group as early as 7 days post treatment (**Figure 1A–B**), and the therapeutic effects were sustained throughout the study period (p<0.05). No significant differences in ischemic lesion volumes in the PDA-001 treatment groups were detected compared with the FBC-control and vehicle control groups (**Figure 1C**, p>0.05). These data suggest that the optimal number of transplanted PDA-001 cells in this experimental protocol of young adult rats is 4×10^6^ cells. 4×10^6^ and 8×10^6^ PDA-001 cells were then tested in older rats (**Figure 2**). Functional response was measured for each older rat before cell therapy and after cell therapy at 7, 14, 21 and 28 days. The global test using the Generalized Estimating Equation (GEE) was performed to detect the differences in functional recovery among the 3 groups (**Figure 2A–B**). No differences in functional recovery were observed for both PDA-001 treatment groups compared to the vehicle control at early evaluations 7 and 14 days after MCAo. However, significant improvements in functional recovery were observed at 21 and 28 days after MCAo for both PDA-001 treatment groups (4×10^6^ and 8×10^6^ cells, p<0.05, **Figure 2A–B**).

No significant differences of ischemic lesion volumes in the PDA-001 treatment groups were detected compared with control group in older rats (p>0.05, **Figure 2C**). Thus, functional outcome improvement after PDA-001 treatment likely results from a neurorestorative effect rather than a neuroprotective effect, when PDA-001 treatment is administered 24 hours after MCAo.

### Endothelial cell proliferation, vascular density and perimeter in the IBZ

To test for the neurorestorative processes which could underlie the PDA-001 induced functional improvements, immunohistochemical analysis was performed.

Enlarged and thin-walled vessels, containing BrdU immunoreactive positive endothelial cells are indicative of angiogenesis [31]. BrdU positive endothelial cells within a total of 10 enlarged and thin walled vessels located in the boundary area of the ischemic lesion were counted in each section.

**Figure 3:**
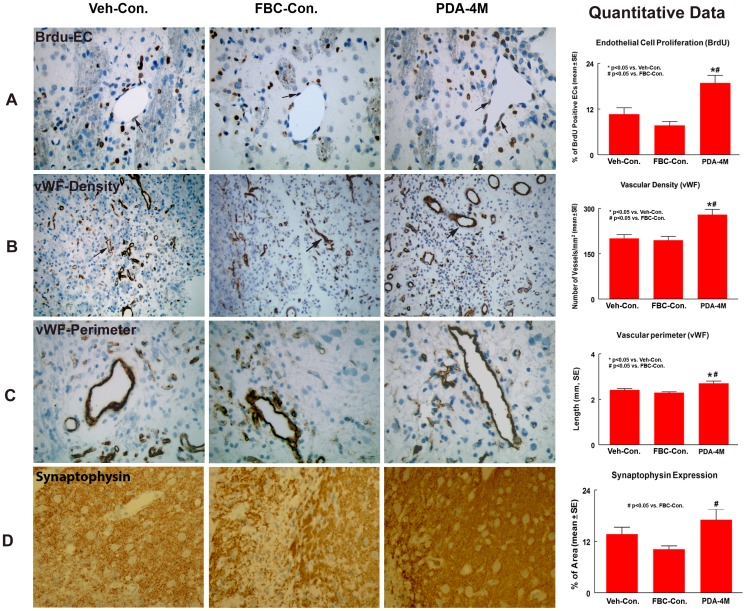
PDA-001 cell treatment promotes endothelial cell proliferation, vascular density and perimeter and increases synaptophysin expression in young adult rats after MCAo. Panels **A–C** show that PDA-001 treatment significantly increased endothelial cell proliferation (**A**), vascular density (**B**) and vascular perimeter (**C**) in the ischemic boundary zone (IBZ) in young adult rats. Panel **D** shows that PDA-001 treatment significantly increased synaptophysin expression in IBZ in young adult rats. Error bars represent standard error of the mean (mean±SE).

**Figure 4:**
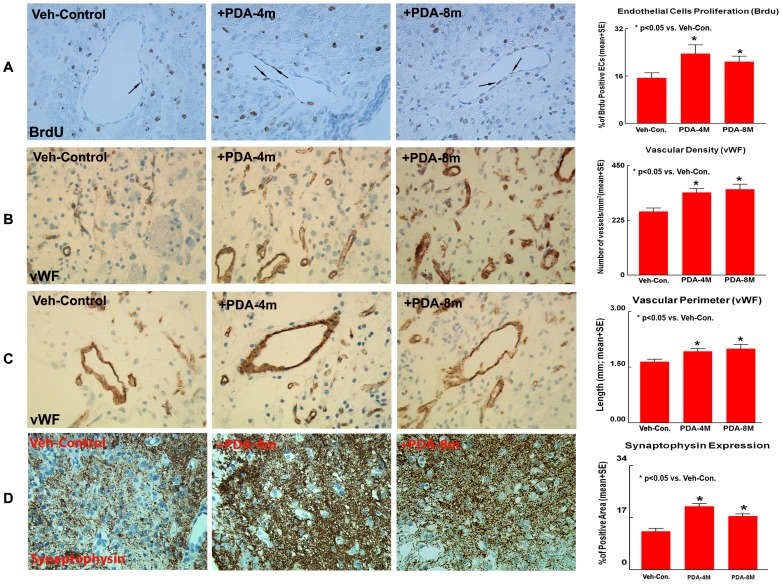
PDA-001 cell treatment promotes endothelial cell proliferation, vascular density and perimeter and increases synaptophysin expression in older rats after MCAo. Panels **A–C** show that PDA-001 treatment significantly increased endothelial cell proliferation (**A**), vascular density (**B**) and vascular perimeter (**C**) in the IBZ in older rats. Panel **D** shows that PDA-001 treatment significantly increased synaptophysin expression in IBZ in older rats. Error bars represent standard error of the mean (mean±SE).

In young adult rats, treatment with 4×10^6^ PDA-001 cells, significantly increased the number of BrdU immunoreactive endothelial cells in the IBZ compared with the FBC and vehicle control groups (**Figure 3A**, p<0.05). In older rats, a significant increase in endothelial cell proliferation in the IBZ was observed in both PDA-001 treatment groups (4×10^6^ and 8×10^6^ cells, **Figure 4A**, p<0.05).

Immunostaining for vWF expression was performed to measure changes in vascular perimeter and density after PDA-001 treatment. In young adult rats, treatment with 4×10^6^ PDA-001 cells significantly increased the vascular density and perimeter in the ischemic brain compared to FBC and vehicle control groups (**Figure 3B–C**, p<0.05).

In older rats, both PDA-001 treatment groups (4×10^6^ and 8×10^6^ cells) showed significant increase in vascular density and perimeter in the IBZ (**Figure 4B–C**, p<0.05). The percentage of BrdU positive endothelial cells was positively and significantly correlated with vascular density (r = 0.47, p = 0.001).

These data suggest that PDA-001 treatment has a positive influence on endothelial proliferation, vascular density and perimeter in the ischemic brain in both young adult and older rats.

### Synaptophysin expression in the IBZ

To test PDA-001 cell treatment effects on synaptic plasticity, synaptophysin immunostaining was performed. An increase in synaptophysin expression in the IBZ suggests enhanced presynaptic plasticity and synaptogenesis [32]. In young adult rats, treatment with 4×10^6^ PDA-001 cells significantly increased synaptophysin expression in the IBZ compared to FBC control (**Figure 3D**, p<0.05).

In older rats, both PDA-001 treatment groups (4×10^6^ and 8×10^6^ cells) also showed a significant increase in synaptophysin expression in the IBZ (**Figure 4D**, p>0.05). We found a marginal correlation (p = 0.051) between the improvement in the adhesive-removal test and synaptophysin expression (r = −0.29). These data suggest that functional benefits derived from PDA-001 treatment of stroke in both young adult and older rats may be related to synaptic plasticity in the ischemic brain.

## Discussion

Our data demonstrate that treatment of stroke with PDA-001, when administered 24 hours after MCAo, improves functional outcome as measured by adhesive-removal test, mNSS and foot-fault test. This beneficial effect is observed in both young adult and older rats.

In young adult rats, the optimal number of transplanted PDA-001 cells is 4×10^6^. A similar optimal number of transplanted cells was observed in a previous study when PDA-001 were administered 4 hours after MCAo [19]. Treatment with a lower (1×10^6^) or a higher (8×10^6^) number of cells is not effective. In addition, our data shows that in older rats, treatment with both 4×10^6^ and 8×10^6^ PDA-001 cells improve functional outcome. The data show that when young rats are treated with PDA-001 cells, significant improvement in functional recovery is observed as early as 7 days post MCAo. However, in treated older rats, significant improvement in functional recovery is delayed and starts at 21 days after MCAo. This is consistent with other published studies, e.g., two previous studies [5], [6] testing sildenafil in young and aged rats showed that the therapeutic response was weaker and delayed in older rats compared to young rats. Thus, brain repair/remodeling processes seem to be age dependent.

In a previous study, we demonstrated that PDA-001 therapy is neuroprotective when administered 4 hours after MCAo as measured by reduction in the ischemic lesion volume and improvement in functional outcome [19]. In the current study, we found that treatment of stroke with PDA-001, when administered 24 hours after stroke in young adult and older rats, has no effect on the volume of cerebral infarction. Thus, functional outcome after PDA-001 treatment when administered 24 hours after stroke, likely results from neurorestorative effect rather than a neuroprotective effect.

Additionally, functional improvement after PDA-001 cell treatment is accompanied by a significant increase in endothelial proliferation, vascular density and perimeter and an increased expression of synaptophysin.

### PDA-001 treatment increases endothelial cell proliferation, vascular density and perimeter

The cerebral vascular system mainly develops through angiogenesis [33]. The adult brain vascular system is stable under normal conditions but is activated in response to pathological conditions including stroke [34]. In rodent models of stroke, capillary sprouting in the brain is initiated at the border of the infarct, and new vessels develop in the ischemic boundary zone [31], [35].

Regulation of cerebral blood flow is critical for the maintenance of neural function [36]. Stroke patients with greater cerebral blood vessel density appear to make better progress and survive longer than patients with lower vascular density [37]–[39]. Additionally, functional improvement in animal stroke models has been associated with increased angiogenesis [40]. Our data demonstrate that PDA-001 treatment promotes endothelial cell proliferation, increases vessel perimeter and density and improves functional recovery in both young adult and older rats after stroke. This suggests that PDA-001 treatment may enhance recovery after stroke through modulation of the brain vascular system.

### PDA-001 treatment increases synaptophysin expression

Synaptic plasticity is an important mediator of functional recovery following brain injury [41], [42]. Functional alterations in motor cortex organization are accompanied by changes in dendritic and synaptic structure [42], [43]. Cortical stimulation promotes synaptic plasticity which is correlated with functional improvements [44]. Synaptophysin in a pre-synaptic marker and increased levels of synaptophysin are observed during neuroanatomical remodeling and neural development, and are indicative of synaptic plasticity. Neurorestorative treatments of stroke increase synaptic plasticity in the ischemic boundary zone [45], [46], as evidenced by increased expression of synaptic proteins such as synaptophysin and growth-associated protein 43 [47]. PDA-001 treatment in both young adult and older rat stroke models is associated with increased synaptophysin expression suggesting that enhanced synaptic plasticity may also contribute to the observed functional improvement.

In summary, PDA-001 treatment improves functional outcome in the rat MCAo model in young as well as older adult rats when administered 24 hours after stroke. Increased vascular density and synaptic plasticity may underlie the neurorestorative effects of PDA-001 therapy.

## References

[1] Takahashi K, Yasuhara T, Shingo T, Muraoka K, Kameda M, et al (2008) Embryonic neural stem cells transplanted in middle cerebral artery occlusion model of rats demonstrated potent therapeutic effects, compared to adult neural stem cells. Brain Res 1234: 172–182. 18703033 10.1016/j.brainres.2008.07.086

[2] Mochizuki N, Takagi N, Kurokawa K, Onozato C, Moriyama Y, et al (2008) Injection of neural progenitor cells improved learning and memory dysfunction after cerebral ischemia. Exp Neurol 211: 194–202. 18346733 10.1016/j.expneurol.2008.01.027

[3] Chen J, Sanberg PR, Li Y, Wang L, Lu M, et al (2001) Intravenous administration of human umbilical cord blood reduces behavioral deficits after stroke in rats. Stroke 32: 2682–2688. 11692034 10.1161/hs1101.098367

[4] Zacharek A, Shehadah A, Chen J, Cui X, Roberts C, et al Comparison of bone marrow stromal cells derived from stroke and normal rats for stroke treatment. Stroke 41: 524–530. 20056925 10.1161/STROKEAHA.109.568881 PMC2847444

[5] Chen J, Li Y, Wang L, Zhang Z, Lu D, et al (2001) Therapeutic benefit of intravenous administration of bone marrow stromal cells after cerebral ischemia in rats. Stroke 32: 1005–1011. 11283404 10.1161/01.str.32.4.1005

[6] Li Y, Chen J, Wang L, Lu M, Chopp M (2001) Treatment of stroke in rat with intracarotid administration of marrow stromal cells. Neurology 56: 1666–1672. 11425931 10.1212/wnl.56.12.1666

[7] Rempe DA, Kent TA (2002) Using bone marrow stromal cells for treatment of stroke. Neurology 59: 486–487. 12196638 10.1212/wnl.59.4.486

[8] Yoon YS, Lee N, Scadova H (2005) Myocardial regeneration with bone-marrow-derived stem cells. Biol Cell 97: 253–263. 15762847 10.1042/BC20040099

[9] Kinnaird T, Stabile E, Burnett MS, Shou M, Lee CW, et al (2004) Local delivery of marrow-derived stromal cells augments collateral perfusion through paracrine mechanisms. Circulation 109: 1543–1549. 15023891 10.1161/01.CIR.0000124062.31102.57

[10] Li Y, Chopp M (2009) Marrow stromal cell transplantation in stroke and traumatic brain injury. Neurosci Lett 456: 120–123. 19429146 10.1016/j.neulet.2008.03.096 PMC3359793

[11] Pittenger MF, Mackay AM, Beck SC, Jaiswal RK, Douglas R, et al (1999) Multilineage potential of adult human mesenchymal stem cells. Science 284: 143–147. 10102814 10.1126/science.284.5411.143

[12] Rao MS, Mattson MP (2001) Stem cells and aging: expanding the possibilities. Mech Ageing Dev 122: 713–734. 11322994 10.1016/s0047-6374(01)00224-x

[13] He S, Khan J, Gleason J, Eliav E, Fik-Rymarkiewicz E, et al (2013) Placenta-derived adherent cells attenuate hyperalgesia and neuroinflammatory response associated with perineural inflammation in rats. Brain Behav Immun 27: 185–192. 23103445 10.1016/j.bbi.2012.10.015

[14] Li X, Ling W, Pennisi A, Wang Y, Khan S, et al (2011) Human placenta-derived adherent cells prevent bone loss, stimulate bone formation, and suppress growth of multiple myeloma in bone. Stem Cells 29: 263–273. 21732484 10.1002/stem.572 PMC3175303

[15] Mayer L, Pandak WM, Melmed GY, Hanauer SB, Johnson K, et al (2013) Safety and tolerability of human placenta-derived cells (PDA001) in treatment-resistant crohn's disease: a phase 1 study. Inflamm Bowel Dis 19: 754–760. 23429460 10.1097/MIB.0b013e31827f27df PMC4272923

[16] Nichols RC, Huh SN, Henderson RH, Mendenhall NP, Flampouri S, et al (2011) Proton radiation therapy offers reduced normal lung and bone marrow exposure for patients receiving dose-escalated radiation therapy for unresectable stage iii non-small-cell lung cancer: a dosimetric study. Clin Lung Cancer 12: 252–257. 21726825 10.1016/j.cllc.2011.03.027

[17] Kranz A, Wagner DC, Kamprad M, Scholz M, Schmidt UR, et al (2010) Transplantation of placenta-derived mesenchymal stromal cells upon experimental stroke in rats. Brain Res 1315: 128–136. 20004649 10.1016/j.brainres.2009.12.001

[18] Yu S, Tajiri N, Franzese N, Franzblau M, Bae E, et al (2013) Stem Cell-Like Dog Placenta Cells Afford Neuroprotection against Ischemic Stroke Model via Heat Shock Protein Upregulation. PLoS One 8: e76329. 24086730 10.1371/journal.pone.0076329 PMC3783428

[19] Chen J, Shehadah A, Pal A, Zacharek A, Cui X, et al (2012) Neuroprotective Effect of Human Placenta-derived Cell Treatment of Stroke in Rats. Cell Transplant 10.3727/096368911X637380 22469567

[20] Zhang L, Zhang RL, Wang Y, Zhang C, Zhang ZG, et al (2005) Functional recovery in aged and young rats after embolic stroke: treatment with a phosphodiesterase type 5 inhibitor. Stroke 36: 847–852. 15746452 10.1161/01.STR.0000158923.19956.73

[21] Futrell N, Garcia JH, Peterson E, Millikan C (1991) Embolic stroke in aged rats. Stroke 22: 1582–1591. 1962334 10.1161/01.str.22.12.1582

[22] Ding G, Jiang Q, Li L, Zhang L, Zhang Z, et al (2011) Longitudinal magnetic resonance imaging of sildenafil treatment of embolic stroke in aged rats. Stroke 42: 3537–3541. 21903952 10.1161/STROKEAHA.111.622092 PMC3226838

[23] Chen J, Zacharek A, Li A, Zhang C, Ding J, et al (2006) Vascular endothelial growth factor mediates atorvastatin-induced mammalian achaete-scute homologue-1 gene expression and neuronal differentiation after stroke in retired breeder rats. Neuroscience 141: 737–744. 16730914 10.1016/j.neuroscience.2006.04.042 PMC2791335

[24] Chen H, Chopp M, Welch KM (1991) Effect of mild hyperthermia on the ischemic infarct volume after middle cerebral artery occlusion in the rat. Neurology 41: 1133–1135. 2067642 10.1212/wnl.41.7.1133

[25] Schallert T, Whishaw IQ (1984) Bilateral cutaneous stimulation of the somatosensory system in hemidecorticate rats. Behav Neurosci 98: 518–540. 6539617 10.1037//0735-7044.98.3.518

[26] Hernandez TD, Schallert T (1988) Seizures and recovery from experimental brain damage. Exp Neurol 102: 318–324. 3197789 10.1016/0014-4886(88)90226-9

[27] Barth TM, Grant ML, Schallert T (1990) Effects of MK-801 on recovery from sensorimotor cortex lesions. Stroke 21: III153–157. 2237974

[28] Schallert T, Fleming SM, Leasure JL, Tillerson JL, Bland ST (2000) CNS plasticity and assessment of forelimb sensorimotor outcome in unilateral rat models of stroke, cortical ablation, parkinsonism and spinal cord injury. Neuropharmacology 39: 777–787. 10699444 10.1016/s0028-3908(00)00005-8

[29] Swanson RA, Morton MT, Tsao-Wu G, Savalos RA, Davidson C, et al (1990) A semiautomated method for measuring brain infarct volume. J Cereb Blood Flow Metab 10: 290–293. 1689322 10.1038/jcbfm.1990.47

[30] Lu M, Chen J, Lu D, Yi L, Mahmood A, et al (2003) Global test statistics for treatment effect of stroke and traumatic brain injury in rats with administration of bone marrow stromal cells. J Neurosci Methods 128: 183–190. 12948561 10.1016/s0165-0270(03)00188-2

[31] Zhang ZG, Zhang L, Tsang W, Soltanian-Zadeh H, Morris D, et al (2002) Correlation of VEGF and angiopoietin expression with disruption of blood-brain barrier and angiogenesis after focal cerebral ischemia. J Cereb Blood Flow Metab 22: 379–392. 11919509 10.1097/00004647-200204000-00002

[32] Ujike H, Takaki M, Kodama M, Kuroda S (2002) Gene expression related to synaptogenesis, neuritogenesis, and MAP kinase in behavioral sensitization to psychostimulants. Ann N Y Acad Sci 965: 55–67. 12105085 10.1111/j.1749-6632.2002.tb04151.x

[33] Risau W (1998) Development and differentiation of endothelium. Kidney Int Suppl 67: S3–6. 10.1046/j.1523-1755.1998.06701.x 9736244

[34] Greenberg DA, Jin K (2005) From angiogenesis to neuropathology. Nature 438: 954–959. 16355213 10.1038/nature04481

[35] Garcia JH, Cox JV, Hudgins WR (1971) Ultrastructure of the microvasculature in experimental cerebral infarction. Acta Neuropathol 18: 273–285. 4999175 10.1007/BF00688441

[36] Pratt PF, Medhora M, Harder DR (2004) Mechanisms regulating cerebral blood flow as therapeutic targets. Curr Opin Investig Drugs 5: 952–956. 15503650

[37] Cramer SC, Nelles G, Benson RR, Kaplan JD, Parker RA, et al (1997) A functional MRI study of subjects recovered from hemiparetic stroke. Stroke 28: 2518–2527. 9412643 10.1161/01.str.28.12.2518

[38] Krupinski J, Kaluza J, Kumar P, Kumar S, Wang JM (1994) Role of angiogenesis in patients with cerebral ischemic stroke. Stroke 25: 1794–1798. 7521076 10.1161/01.str.25.9.1794

[39] Cramer SC, Chopp M (2000) Recovery recapitulates ontogeny. Trends Neurosci 23: 265–271. 10838596 10.1016/s0166-2236(00)01562-9

[40] Zhang ZG, Chopp M (2009) Neurorestorative therapies for stroke: underlying mechanisms and translation to the clinic. Lancet Neurol 8: 491–500. 19375666 10.1016/S1474-4422(09)70061-4 PMC2727708

[41] Kolb B (1999) Synaptic plasticity and the organization of behaviour after early and late brain injury. Can J Exp Psychol 53: 62–76. 10389490 10.1037/h0087300

[42] Nudo RJ, Plautz EJ, Frost SB (2001) Role of adaptive plasticity in recovery of function after damage to motor cortex. Muscle Nerve 24: 1000–1019. 11439375 10.1002/mus.1104

[43] Bohotin CR, Badescu M, Popescu DN, Bohotin V (2004) Motor cortex plasticity–from physiology to clinical neurology. Rom J Physiol 41: 99–108. 15984660

[44] Adkins DL, Hsu JE, Jones TA (2008) Motor cortical stimulation promotes synaptic plasticity and behavioral improvements following sensorimotor cortex lesions. Exp Neurol 212: 14–28. 18448100 10.1016/j.expneurol.2008.01.031 PMC3018150

[45] Cui X, Chopp M, Zacharek A, Roberts C, Buller B, et al (2010) Niacin treatment of stroke increases synaptic plasticity and axon growth in rats. Stroke 41: 2044–2049. 20671245 10.1161/STROKEAHA.110.589333 PMC2932778

[46] Chen J, Li Y, Wang L, Lu M, Chopp M (2002) Caspase inhibition by Z-VAD increases the survival of grafted bone marrow cells and improves functional outcome after MCAo in rats. J Neurol Sci 199: 17–24. 12084437 10.1016/s0022-510x(02)00075-8

[47] Stroemer RP, Kent TA, Hulsebosch CE (1998) Enhanced neocortical neural sprouting, synaptogenesis, and behavioral recovery with D-amphetamine therapy after neocortical infarction in rats. Stroke 29: 2381–2393 discussion 2393-2385. 9804653 10.1161/01.str.29.11.2381

